# Supplementary material for: Unique referral system contributes to long-term net clinical benefits in patients undergoing secondary prevention therapy after percutaneous coronary intervention
Source: PLoS One. 2020 Nov 19;15(11):e0242707. doi: 10.1371/journal.pone.0242707 (PMC7676744; doi:10.1371/journal.pone.0242707)
Supplement: Attachment — Submitted filename: Response to Reviewers.docx [file pone.0242707.s003.docx]

'Response to Reviewers'

PONE-D-20-25225

Unique referral system contributes to long-term net clinical benefits in patients undergoing secondary prevention therapy after percutaneous coronary intervention

PLOS ONE

Dear Dr. KAGEYAMA,

Thank you for submitting your manuscript to PLOS ONE. After careful consideration, we feel that it has merit but does not fully meet PLOS ONE’s publication criteria as it currently stands. Therefore, we invite you to submit a revised version of the manuscript that addresses the points raised during the review process.

Please submit your revised manuscript by Oct 22 2020 11:59PM. If you will need more time than this to complete your revisions, please reply to this message or contact the journal office at plosone@plos.org. When you're ready to submit your revision, log on to https://www.editorialmanager.com/pone/ and select the 'Submissions Needing Revision' folder to locate your manuscript file.

Please include the following items when submitting your revised manuscript:

A rebuttal letter that responds to each point raised by the academic editor and reviewer(s). You should upload this letter as a separate file labeled 'Response to Reviewers'.

A marked-up copy of your manuscript that highlights changes made to the original version. You should upload this as a separate file labeled 'Revised Manuscript with Track Changes'.

An unmarked version of your revised paper without tracked changes. You should upload this as a separate file labeled 'Manuscript'.

If you would like to make changes to your financial disclosure, please include your updated statement in your cover letter. Guidelines for resubmitting your figure files are available below the reviewer comments at the end of this letter.

If applicable, we recommend that you deposit your laboratory protocols in protocols.io to enhance the reproducibility of your results. Protocols.io assigns your protocol its own identifier (DOI) so that it can be cited independently in the future. For instructions see: http://journals.plos.org/plosone/s/submission-guidelines#loc-laboratory-protocols

We look forward to receiving your revised manuscript.

Kind regards,

Yoshihiro Fukumoto

Academic Editor

PLOS ONE

Journal Requirements:

When submitting your revision, we need you to address these additional requirements.

1. Please ensure that your manuscript meets PLOS ONE's style requirements, including those for file naming. The PLOS ONE style templates can be found at

https://journals.plos.org/plosone/s/file?id=wjVg/PLOSOne_formatting_sample_main_body.pdf　and

https://journals.plos.org/plosone/s/file?id=ba62/PLOSOne_formatting_sample_title_authors_affiliations.pdf

We have read and understood your journal’s policies, and we believe that neither the manuscript nor the study violates any of these. The manuscript has been rechecked, and the necessary changes have been made in accordance with the reviewers’ suggestions. The changes in the manuscript are highlighted in yellow, and the responses to all comments have been prepared and attached herewith.

2. Please include captions for your Supporting Information files at the end of your manuscript, and update any in-text citations to match accordingly. Please see our Supporting Information guidelines for more information: http://journals.plos.org/plosone/s/supporting-information.

We do not have any additional supporting information files.

[Note: HTML markup is below. Please do not edit.]

Reviewers' comments:

Reviewer's Responses to Questions

Comments to the Author

1. Is the manuscript technically sound, and do the data support the conclusions?

The manuscript must describe a technically sound piece of scientific research with data that supports the conclusions. Experiments must have been conducted rigorously, with appropriate controls, replication, and sample sizes. The conclusions must be drawn appropriately based on the data presented.

Reviewer #1: Yes

Reviewer #2: Yes

2. Has the statistical analysis been performed appropriately and rigorously?

Reviewer #1: Yes

Reviewer #2: I Don't Know

3. Have the authors made all data underlying the findings in their manuscript fully available?

The PLOS Data policy requires authors to make all data underlying the findings described in their manuscript fully available without restriction, with rare exception (please refer to the Data Availability Statement in the manuscript PDF file). The data should be provided as part of the manuscript or its supporting information, or deposited to a public repository. For example, in addition to summary statistics, the data points behind means, medians and variance measures should be available. If there are restrictions on publicly sharing data—e.g. participant privacy or use of data from a third party—those must be specified.

Reviewer #1: Yes

Reviewer #2: No

4. Is the manuscript presented in an intelligible fashion and written in standard English?

PLOS ONE does not copyedit accepted manuscripts, so the language in submitted articles must be clear, correct, and unambiguous. Any typographical or grammatical errors should be corrected at revision, so please note any specific errors here.

Reviewer #1: Yes

Reviewer #2: Yes

5. Review Comments to the Author

Reviewer #1: This single-center, retrospective observational study evaluated the impact and long-term benefits of unique referral system to 3658 consecutive patients with ischemic heart disease undergoing secondary prevention therapy after PCI. The authors showed that the occurrence of net adverse clinical events was significantly lower in the referral system group than in the conventional follow-up group even after propensity-score matching. This study is interesting and provides useful information although this is a single-center, retrospective observational study. However, this study needs to be improved in some points.

1. As the authors stated in the limitation section of the discussion, baseline patient characteristics are too heterogeneous. Even after propensity-score matching, some risk factors had statistically significant. The authors should discuss the significance of the multicenter, prospective study in this topic.

Thank you for this important suggestion. I have added the importance of a future multicenter prospective study to the limitations paragraph of the Discussion section (lines 371-372).

2. Regarding the Figures 3 and 4, when was the start of follow up (ie, 0 day). The authors should set the start date of the follow up to the registration date not to PCI date. The Kaplan-Meier curve demonstrated that the event rate between 2 groups seems to have the difference within 1 years. It might be a vias if the event rate has the significant difference before the registration. The authors should clearly state this point in the Method and Result sections.

Thank you for your valuable input. The time between the latest PCI and enrollment in the unique referral system varied among cases, and in actual practice, some patients can be enrolled in the referral system after the event. In this study, however, patients who experienced events before enrollment were classified into the conventional follow-up group. Therefore, the event rate before registration (and after the index PCI) was not known for either group. However, the follow-up term was significantly longer in the conventional follow-up group than in the unique referral system group. The median duration from index PCI to registration in the unique referral system group was 60 days (IQR 2-299). The start of conventional follow-up was within 90 days after the index PCI. Therefore, for propensity score matching, we only included patients from the unique referral group who had less than 100 days between the index PCI and the registration. We also excluded events occurring before discharge. These aspects have been clarified in the Materials and Methods section (lines 85, 90-91, 181-185).

Reviewer #2: This manuscript investigated clinical benefits of the audit referral system which has been built for coordinate care between general practitioners and cardiologists.

The authors found that their referral system contributed to better long-term clinical benefits for the secondary prevention of ischemic heart disease after percutaneous coronary intervention than conventional follow-up.

This reviewer has the following comments:

- Please provide the detail of MACCE.

If the majority of the overall events are revascularization, more detailed information on lesion background (e.g. complexity) and procedural information at the index PCI should be provided.

Thank you for your suggestion. Revascularization accounted for 65.4% of MACCE (n = 538), and CHF accounted for 25.9% (n = 213). MI accounted for 6.5% (n = 54). Ventricular arrhythmia accounted for 2.9% (n = 24). Each factor did not have any statistically significant differences between the conventional follow-up group and the referral system group. Regarding the lesion complexity at the index PCI, the incidence of type B2/C lesions was 53.3%. The average number of target lesions was 1.22. We have added all relevant details to Table 1 and the Results section (lines 234–237).

- Definition of each item in MACCE should be explained, such as MI, AP, ventricular arrhythmia, congestive heart failure, and peripheral artery disease.

Thank you for your suggestion. I have added the relevant definitions in the Materials and Methods section (lines 149-157). MI was classified as type 1 and type 2 MI according to the Fourth Universal Definition of Myocardial Infarction (Thygesen K, Alpert JS, Jaffe AS, Chaitman BR, Bax JJ, Morrow DA, White HD; Executive Group on behalf of the Joint European Society of Cardiology (ESC)/American College of Cardiology (ACC)/American Heart Association (AHA)/World Heart Federation (WHF) Task Force for the Universal Definition of Myocardial Infarction. Fourth Universal Definition of Myocardial Infarction (2018). J Am Coll Cardiol. 2018 Oct 30;72(18):2231-2264). AP was defined as angina necessitating hospitalization, with confirmation of ischemia by angiography and/or scintigraphy. Ventricular arrhythmia was defined as ventricular tachycardia or ventricular fibrillation detected by 12-lead electrocardiography and warranting hospitalization and/or antiarrhythmic drugs. Congestive heart failure was defined as heart failure necessitating hospitalization, oxygenation, and diuretics. Peripheral artery disease was defined as arteriosclerosis obliterans necessitating intervention. Ischemic stroke was defined as stroke necessitating hospitalization and confirmed by head magnetic resonance imaging.

- To confirm the advantages of the referral system over conventional follow-up, the data regarding risk factor control during the follow-up period would be helpful.

Thank you for your suggestion. Unfortunately, we could not obtain details regarding risk factor control for the conventional follow-up group, particularly for patients followed up at outpatient clinics. However, we have added the baseline and latest follow-up data for the unique referral system group in the Results section (lines 219-227).

- The study design is unclear. Particularly, methodology for data collection should be explained clearly. Data were collected every year or at the specific time point?

We apologize for the lack of clarity. Follow-up data were obtained when patients made regular outpatient visits to our hospital at annual referral visits following the Shizuoka IHD referral system, and at non-routine visits after referral from a general practitioner in relation to an event. In order to supplement the missing death events and non-cardiac events, we also performed a prognostic study by analyzing telephone and medical records obtained between March 2019 and May 2020. We have explained this in the Materials and Methods section (lines 136-140).
