# Supplementary material for: Unique referral system contributes to long-term net clinical benefits in patients undergoing secondary prevention therapy after percutaneous coronary intervention
Source: PLoS One. 2020 Nov 19;15(11):e0242707. doi: 10.1371/journal.pone.0242707 (PMC7676744; doi:10.1371/journal.pone.0242707)
Supplement: Attachment — Submitted filename: Response to Reviewers 2.docx [file pone.0242707.s004.docx]

October 29, 2020

Dr. Joerg Heber

Editor-in-Chief

*PLoS ONE*

Dear Dr. Heber:

We wish to re-submit our manuscript, with ID PONE-D-20-25225R1, titled, “Unique referral system contributes to long-term net clinical benefits in patients undergoing secondary prevention therapy after percutaneous coronary intervention”.

We appreciate the time and effort you and the reviewers have dedicated to providing thoughtful suggestions and valuable insights. Our manuscript has greatly benefited from your valuable feedback.

We have revised our manuscript and made the necessary changes reflecting the detailed suggestions you have graciously provided. Our responses are given in a point-by-point manner below and corresponding changes to the manuscript are highlighted in yellow and in tracks. We hope that the edits and the responses we have prepared and provided below satisfactorily address the concerns you and the reviewers have noted.

We look forward to working with you and the reviewers to move this manuscript closer to publication in *PLoS ONE*.

Respectfully,

Shigetaka Kageyama

Shizuoka City Shizuoka Hospital,

Department of Cardiology, Shizuoka, Japan

[shigenewf@yahoo.co.jp](mailto:shigenewf@yahoo.co.jp)

**Point-by-point responses**

Reviewers' comments:

Reviewer #1: The authors almost responded to the reviewer’s remarks in detail. No additional comments would be needed.

Response: Thank you for the positive feedback.

Reviewer #2: I feel that the manuscript has been revised well.

Response: Thank you for the positive feedback.

Comment 1: Now, I have one comment. - Please discuss why the clinical event occurrence was favorable in the referral system group, even though some risk factor control has got worse slightly.

Response: Thank you for your valuable comments. As you have correctly observed, risk factor control in the referral group only showed an improvement in LDL-C levels and exacerbation in HbA1c and blood pressure control compared to baseline. Despite the worsening of the latter two factors, the incidence of clinical events was lower in the referral group than in the conventional follow-up group. This discrepancy can be explained by the fact that our unique referral system lowered the threshold for general practitioners in recommending their patients to consult with a cardiologist. In turn, this could have potentially led to a net clinical benefit for the patient as it was easier to consult with other specialists in the general hospital, such as cerebrovascular disease and bleeding events specialists, using the referral to cardiologists as a starting point. Another potential reason is the fact that these factors do not perhaps pose the same level of risk of developing a clinical event. Many large clinical trials have reported that cardiovascular events are directly correlated with LDL-C levels in the secondary prevention of ischemic heart disease; however, it is possible that HbA1c levels or blood pressure are inferior risk indicators for cardiovascular events compared to LDL-C levels. In a large population-based prospective study in Japan, the risk of coronary artery disease was significantly higher in the hypertensive group (systolic blood pressure of 135 mm Hg or higher, diastolic blood pressure of 85 mm Hg or higher, or those taking antihypertensive drugs) than in a normotensive group. However, the risk probably could not have counteracted the impact of the improved prognostic effect of lower LDL-C in this study population. We have added these points to the Discussion section of the revised manuscript.

Discussion, p.26, lines 343-358:

“In this study, risk factor control in the referral group only showed an improvement in LDL-C levels and an exacerbation in HbA1c and blood pressure control compared to baseline. Despite the worsening of the latter two factors, the incidence of clinical events was still lower in the referral group than in the conventional follow-up group. This discrepancy can be explained by the fact that our unique referral system lowered the threshold for general practitioners in recommending their patients to consult with a cardiologist. In turn, this could have potentially led to a net clinical benefit for the patient since it was easier to consult with other specialists in the general hospital, such as cerebrovascular disease and bleeding event specialists, using the referral to the cardiologist as a starting point. Another potential reason is the fact that these factors perhaps do not pose the same level of risk of developing a clinical event. Many large clinical trials have reported that cardiovascular events are directly correlated with LDL-C levels in the secondary prevention of ischemic heart disease [4, 16]; however, it is possible that HbA1c levels or blood pressure are inferior risk indicators for cardiovascular events compared to LDL-C levels [19]. In a large population-based prospective study in Japan, the risk of coronary artery disease was significantly higher in the hypertensive group than in the normotensive group [20]. However, the risk probably could not have counteracted the impact of the improved prognostic effect of lower LDL-C in this study population.”
